# Unravelling the dynamical origin of below- and near-threshold harmonic generation of H_2_^+^ in an intense NIR laser field

**DOI:** 10.1038/srep37774

**Published:** 2016-11-24

**Authors:** John Heslar, Shih-I. Chu

**Affiliations:** 1 Center for Quantum Science and Engineering, and Center for Advanced Study in Theoretical Sciences, Department of Physics, National Taiwan University, Taipei 10617, Taiwan; 2 Department of Chemistry, University of Kansas, Lawrence, Kansas 66045, USA

## Abstract

Recently, the study of near- and below- threshold regime harmonics as a potential source of intense coherent vacuum-ultraviolet radiation has received considerable attention. However, the dynamical origin of these lower harmonics, particularly for the molecular systems, is less understood and largely unexplored. Here we perform the first fully *ab initio* and high precision 3D quantum study of the below- and near-threshold harmonic generation of 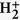 molecules in an intense 800-nm near-infrared (NIR) laser field. Combining with a synchrosqueezing transform of the quantum time-frequency spectrum and an extended semiclassical analysis, we explore in-depth the roles of various quantum trajectories, including short- and long trajectories, multiphoton trajectories, resonance-enhanced trajectories, and multiple rescattering trajectories of the below- and near- threshold harmonic generation processes. Our results shed new light on the dynamical origin of the below- and near-threshold harmonic generation and various quantum trajectories for diatomic molecules for the first time.

High-order-harmonic generation (HHG) is a fundamental atomic and molecular process in strong laser fields that continues to receive considerable attention and it plays a crucial role in the development of ultrafast science and technology^1^,^2^. Significant application of the HHG technology includes the generation of ultrashort attosecond pulses^3^,^4^,^5^ and ultrafast molecular imaging^6^,^7^, etc., to name only a few. The availability of the attosecond pulse further allows the direct detection and control of the electronic dynamics in atoms, molecules and condensed matter systems^2^,^3^,^5^,^6^,^7^,^8^,^9^,^10^. The HHG spectrum is characterized by a rapid drop in intensity at low orders followed by a broad plateau and then a cutoff at approximately *I*_*p*_ + 3.17 *U*_*p*_, where *I*_*p*_ is the ionization potential of the atom/molecule and *U*_*p*_ is the ponderomotive potential. The general pattern of the HHG power spectrum for harmonics above the ionization threshold *I*_*p*_ can be qualitatively explained by means of the 3-step model^11^,^12^. According to this model, the atom (molecule) is first ionized by the laser field by either tunneling or multiphoton ionization. The ionized electron is accelerated by the laser field and acquires some additional energy. Oscillating in the field, the electron can return to the core and undergoes a recollision. As a result, a recombination can occur, and the extra electron energy is converted into a photon of the harmonic radiation. It is well known that the electron trajectories fall into two families, namely short and long trajectories, depending on the ionization and returning time of the electrons. Since molecules are more complex systems than atoms, due to the extra internuclear degree of freedom, the rescattering dynamical behavior of the electron molecular orbits are topologically different from that of the atoms and is still not fully understood. For example, for diatomic molecules, besides the recollision with the parent ion core which resembles the single atom case, the recollision can also occur with the other nucleus.

In the past, major attention was focused on the HHG regime well above the ionization threshold where the semiclassical three-step model^11^,^12^ and strong field approximation (SFA)^13^ are effective to explain the process. More recently considerable attention has been paid to the near- and below-threshold regimes^14^,^15^,^16^,^17^,^18^,^19^,^20^,^21^,^22^ as a potential source of coherent vacuum-ultraviolet radiation^2^. In these lower-energy regimes, the conventional three-step model and the SFA become inadequate, since they neglect the Coulomb potential and the detailed electronic structure of the target atom/molecule. Nevertheless, experimental observations have shed some light on the mechanism for the below- and near-threshold regimes for atoms. Power *et al*.^14^ and Yost *et al*.^15^ suggested that the generation of the below-threshold harmonics is dominated by long trajectories, owing to the influence of the atomic potential. By studying near-threshold HHG from the aligned molecules, Soifer *et al*.^17^ showed that the near-threshold long trajectories belong to the conventional three-step model while the short trajectories stem from multiphoton-driven pathways. As we have recently demonstrated along with the experimentalists^18^, phase-matched below-threshold harmonics can be generated only near the resonance structures of the atomic target. More recently, theoretical investigations by Li *et al*. uncover the subtle electron dynamics for near- and below-threshold harmonic generation in H^23^ and Cs^24^ atoms in strong laser fields.

In this work, we present the first *ab initio* precision study of the below- and near-threshold harmonic generation of the 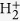 molecule in intense near-infrared laser field by solving the three-dimensional two-centered time-dependent Schrödinger equation (TDSE) accurately and efficiently by means of the time-dependent generalized pseudospectral method (TDGPS)^25^ in prolate spheroidal coordinates. First, we have performed the field-free calculations of the electronic eigenstates of 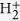 at the equilibrium internuclear separation 2 a.u. The first seven bound states energies with high accuracy are listed (Supplementary Table 1), hence, we do not list all the eigenvalues as they are well-known in the literature [see, e.g., ref. 26]. In addition, we employ a synchrosqueezing transform^27^,^28^,^29^,^30^ (SST) (Supplementary Method) to analyse the time-frequency spectra of the below-, near- and above-threshold HHG of 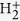. By comparing the SST time-frequency spectra and the extended semiclassical calculations, we unravel for the first time the contributions of the short trajectories, long trajectories, multi-scattering trajectories, “resonant” or bounded trajectories, and “continuum” trajectories in the below-, near- and above-threshold harmonic generation, and demonstrate the features of the spectral dynamical phase for the 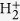 molecule. We find that the multiphoton-dominated processes for the long trajectories only occur when the electrons are scattered off the high part of the combined molecule-field barrier potential (HBP) followed by the absorption of many photons in the below- and near-threshold harmonic generation. In particular, we uncover the short trajectories are multiphoton-dominated and only have one single return, and the tunneling-dominated “continuum” trajectories do not always return to the same hydrogen core the electron was released from. Furthermore, we find in near-threshold harmonic generation, the multi-rescattering trajectories phase at the peak intensity is constant for every optical cycle. These near-threshold multi-rescattering trajectories are the only type that have locked dynamical phases, where the long-trajectories are very sensitive to the laser profile.

## Results

### Harmonic spectra of 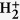


**Figure 1:**
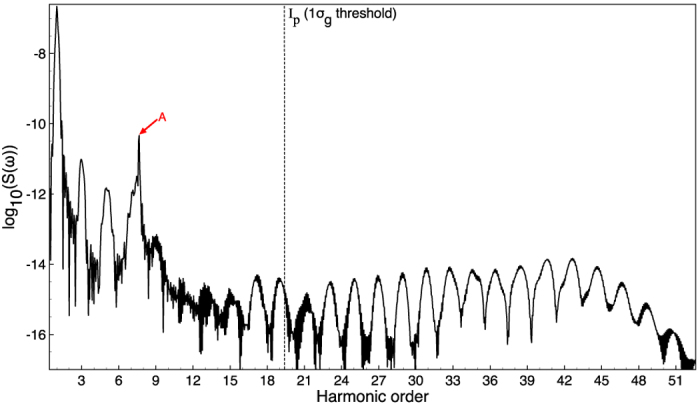
HHG power spectra of 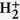. The HHG power spectrum of 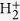 driven by an intense 800-nm (near-infrared) laser pulse with the peak intensity *I* = 2 × 10^14^ W/cm^2^. The black vertical dashed line indicates the corresponding ionization threshold marked by *I*_*p*_ (1*σ*_*g*_). Resonance A corresponds to excitation of the 1*σ*_*u*_ state.

Figure 1 shows the HHG power spectrum of the 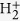 molecule calculated by solving the three-dimensional TDSE with an accurate TDGPS method in an intense near-infrared laser field (Supplementary Method). In the calculation, we adopt a 800-nm near-infrared laser wavelength and the laser pulse shape is similar to the laser pulse reported in recent experiments^18^. The main pulse sits on a pedestal of a weaker field (4% of the peak intensity) which also has a sine-squared shape and duration of 20 optical cycles and the peak intensity is *I* = 2 × 10^14^ W/cm^2^. Thus the stronger field with six optical cycles is preceded by seven optical cycles and also followed by seven optical cycles of the weaker field. The corresponding Keldysh parameter *γ* is equal to 1.12 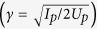, which indicates an intermediate ionization regime (multiphoton ionization is typically characterized by *γ* > 1 while tunneling ionization by *γ* < 1). The molecular ionization potential (1*σ*_*g*_ orbital) of 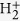 is equal to 1.1026342144949 a.u. (Supplementary Table 1), which coincides with the 19.3 harmonic order for the 800-nm laser pulse. In Fig. 1, we have clearly identified the excited-state resonance peak (red arrow marked by A) near the harmonic 7 (H7), which corresponds to the bound-bound transition from 1*σ*_*g*_–1*σ*_*u*_. The black vertical dashed line shown in Fig. 1 indicates the corresponding ionization threshold marked by *I*_*p*_. For the 800-nm laser wavelength, the harmonic 21 (H21) is just above the ionization threshold.

### SST time-frequency analysis and semiclassical trajectories

To analyse the underlying mechanism from the *ab initio* simulation, we perform the time-frequency analysis of the dipole acceleration for 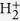 interacting with the applied laser field. In previous studies^27^, several representative time-frequency methods have been compared for the atomic hydrogen system including the short-time Fourier transforms, such as Gabor transform, Wigner-Ville transform^28^ and the SST^24^,^31^, as well as the continuous wavelet transform, the bilinear time-frequency transform and the reallocation method, respectively. They found that both the Gabor and the Morlet transforms are subject to some obscure spectral features arising from a window and that the Wigner-Ville transform is accompanied by interference artifacts, resulting in incomprehensible analysis. Among these methods, only the SST can resolve the intrinsic blurring in the Gabor and the Morlet transforms^29^. As a result, we adopt the SST (Supplementary Method) to explore the characteristic behaviors of harmonic spectra below the ionization threshold, which has successfully depicted chronotaxic systems^30^ and cardiovascular systems^32^.

**Figure 2:**
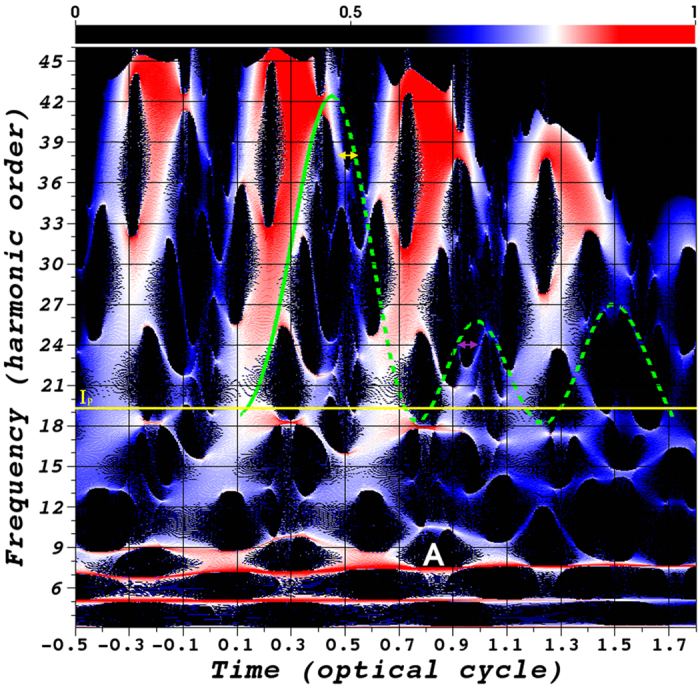
SST time-frequency spectra and semiclassical trajectories. SST time-frequency analysis of the HHG spectra of 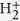. For comparison, the green curves (both solid lines and dashed) indicate the semiclassical result shown in Fig. 3(b) and the yellow solid line indicates the ionization potential *I*_*p*_. For clarity, we only show the semiclassical simulation for the electrons released during one optical cycle preceding the pulse peak. Resonance A corresponds to excitation of the 1*σ*_*u*_ state. The horizontal yellow (purple) arrow is to help guide the eye for the first return long trajectory (second return multirescattering trajectory) in the SST time-frequency analysis and the semiclassical result. The laser parameters used are the same as those in Fig. 1.

**Figure 3:**
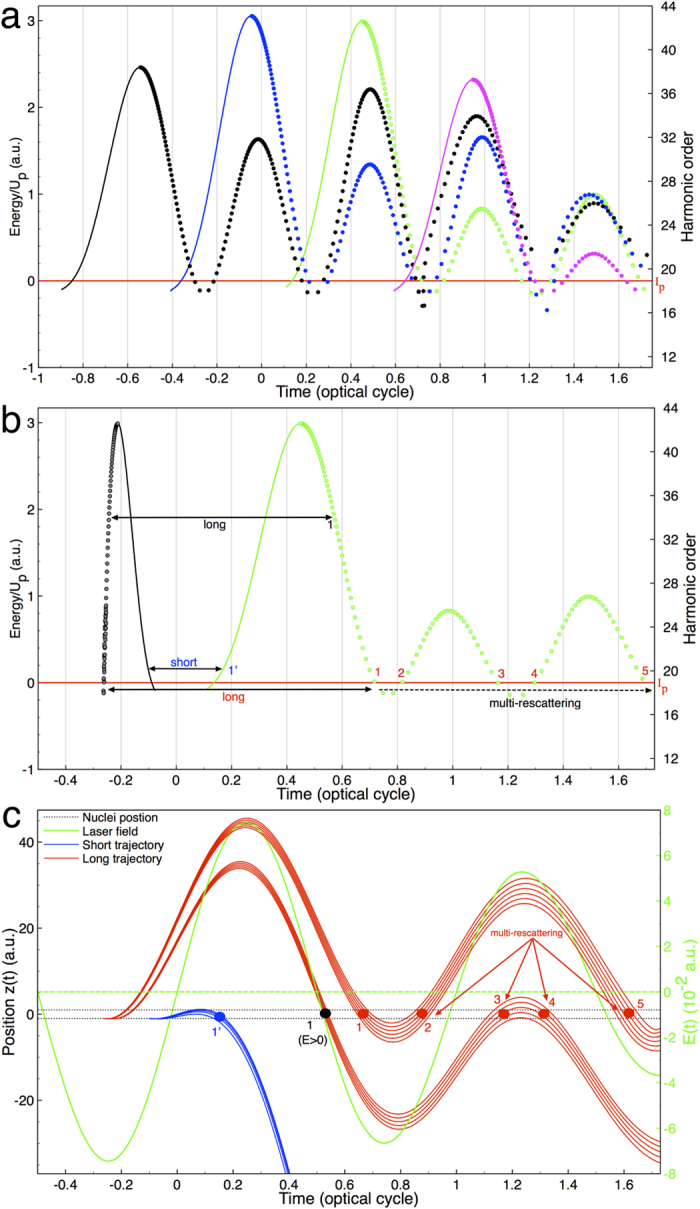
Semiclassical trajectories: Energy, position, and time, and scheme of electron dynamics. (**a**) Semiclassical return energy as a function of several ionization times and return times. The solid and dotted colored lines represent the short and long trajectories, respectively. Here we can see the multi-rescattering of the long trajectories at different return times. (**b**) For clarity, we show the semiclassical return energy as a function of ionization time (black line) and return time (green line) for the electrons released during one optical cycle preceding the pulse peak. Several typical rescatterings are marked by 1’ (blue text) short trajectory, 1 (black text) long trajectory (*E* > 0) and 1 (red text) long trajectory (*E* = 0), and multi-rescattering trajectories are marked by 2–5 (red text). (**c**) Position vs time in below- and near-threshold regions for the corresponding return energies shown in (**b**). The black horizontal dashed line indicates the position of the two hydrogen nuclei (*z* = ±1 a.u.) for 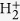 and the colored dots are to help guide the eye for the return times for different trajectories. Here the initial condition is that the electrons with an initial velocity *v*_0_ are released from the left side hydrogen core (*z* = −1 a.u.) along the electronic-field force **F**_*z*_. The green solid line indicates the corresponding laser field, and the laser parameters used are the same as those in Fig. 1.

The time-frequency representation in Fig. 2 shows a periodic repetition of arches comprising the short and long trajectories. It is readily observed that the main contribution to the above-threshold harmonics is due to the short trajectories (dense red color in Fig. 2). The prominent trajectory located near the vicinity of the 7th harmonic is the 1*σ*_*g*_–1*σ*_*u*_ multiphoton resonance-transition of 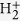. To explore the dynamical role of the quantum trajectories, we extend a standard semiclassical approach suggested independently by Corkum^11^ and Kulander *et al*.^12^ with the inclusion of the molecular potential. Here the electric-field force corresponding to the applied laser field in atomic units is **F**_*z*_ = *E(t*)**e**_**z**_, where **e**_**z**_ is the unit vector in the *z* direction and E(t) is the electric field of the laser pulse. For the laser parameters used, the corresponding Keldysh parameter *γ* is >1 and the multiphoton ionization regime is expected to be dominant, the initial conditions are provided by releasing the electrons with an initial velocity (*v*_0_) to overcome a potential barrier. Therefore, the direction of the initial velocity of electrons is either ‘identical’ or ‘opposite’ with respect to **F**_*z*_. Note that when the direction of the initial velocity of electrons is ‘identical’, the electron gains an extra energy to escape the barrier; when the direction of the initial velocity of electrons is ‘opposite’, the electron is pushed back when leaving the barrier. The semiclassical return energy as a function of the ionization time and return time of the electrons that are released in the first one cycle before the pulse peak for ‘identical’ conditions are presented in Fig. 2 overlaying the SST time-frequency analysis. We indicate the short trajectories (green solid line) and long trajectories (green dashed line) as those in the standard three-step model, as well as the multi-rescattering trajectories [green dashed line (*T* ~ 0.8 − 1.75)]. In Fig. 2, by comparing with the classical calculation, it is clearly seen that the multirescattering trajectory (green dashed line; *T* > 0.7 optical cycles) has strong contributions to the below- and near-threshold harmonic 18–27. Of course, harmonics 18 to 27 have contributions from the short trajectories during subsequent half-cycles. However, it is clearly seen that the multirescattering trajectory has its strong contributions as well. This result is in good agreement with the semiclassical result shown in Supplementary Fig. 1. Particularly, several multirescattering trajectories are superposed at around 0.7–1.75 optical cycles, which contribute to the generation of the below- and near-threshold harmonics. To explore the intricate structures in the below-, near-, and above-threshold generation, we show the semiclassical return energy as a function of several ionization times and return times in Fig. 3a and b. Note that the returning energy includes the kinetic energy and the potential energy, and thus may become negative below the ionization threshold. It is clearly seen that the HHG originated from two quantum trajectories, namely, the short trajectories (the higher harmonics are emitted after the lower ones) and the long trajectories (the higher harmonics are emitted before the lower ones). In Fig. 3a and b, the trajectories that lie between the harmonic 15 and 37 suggest multiple returns of the electron. In Fig. 3b, for clarity, we show the semiclassical return energy as a function of ionization time (black line) and return time (green line) for the electrons released during one optical cycle preceding the pulse peak. We label the short trajectories 1′ and long trajectories 1, as well as the multi-rescattering trajectories 2–5. We find that the long-trajectories electrons associated with the multirescatterings (see the black dashed arrow) contribute to the below- and near-threshold harmonics 17–27, and the corresponding travel time is larger than one optical cycle. The trajectories that are released late and return early are regarded as the short trajectories 1′, while those released early and return late are the long trajectories 1–5. The trajectories 1′ and 1–5 are superimposed in Fig. 2 for the sake of comparison with the SST representation. As shown in the figures, the structures of the SST representation are in agreement with the semiclassical trajectories, although the semiclassical result for the long trajectories are a little broader in time for the first and second return. This can be seen in the range from *T* ~ 0.4 − 0.6 (horizontal yellow arrow), where the semiclassical first return long trajectory (green dashed line) is a little broader than the SST time-frequency analysis. Also, the semiclassical second return multirescattering trajectory (green dashed line) in the range from *T* ~ 0.8 − 1.0 (horizontal purple arrow) is broader in time than seen in the SST representation. Trajectories with ‘identical’ (our initial conditions) and ‘opposite’ initial conditions interchange because the laser field changes sign every half optical cycle. In the quantum mechanical SST representation, trajectories with both ‘identical’ and ‘opposite’ initial conditions all appear in one optical cycle.

The contribution of quantum trajectories to the below-, near-, and above-threshold harmonic generation can be understood according to Figs 2 and 3a–c. For the near-threshold harmonics 17–21, the short trajectories 1′ and the multi-rescattering trajectories 2–5 have the major contributions, while the long trajectories 1 contribute little. Power *et al*.^14^ pointed out recently that long trajectories are the favored pathways in below- and near-threshold harmonic generation. The more recent study by Li *et al*.^23^,^24^,^31^ on atomic systems as well as our current studies of 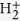 diatomic molecule and SST time-frequency analysis further show that these long trajectories are in fact the multi-rescattering trajectories. In the below-threshold region seen from Fig. 2, extrapolated from the overlaying semiclassical result, we can say short trajectories mainly contribute to harmonics 11–19, and the resonant trajectories contribute to harmonics near 7 (7.65). Note that in Fig. 2, the intensity of the SST time-frequency representation near the 7th order harmonic is strong due to the enhancement of the resonance. The strongest resonant emissions for each optical cycles are located near the laser peak intensity.

### Electron dynamics in below-, near-, and above-threshold HHG

**Figure 4:**
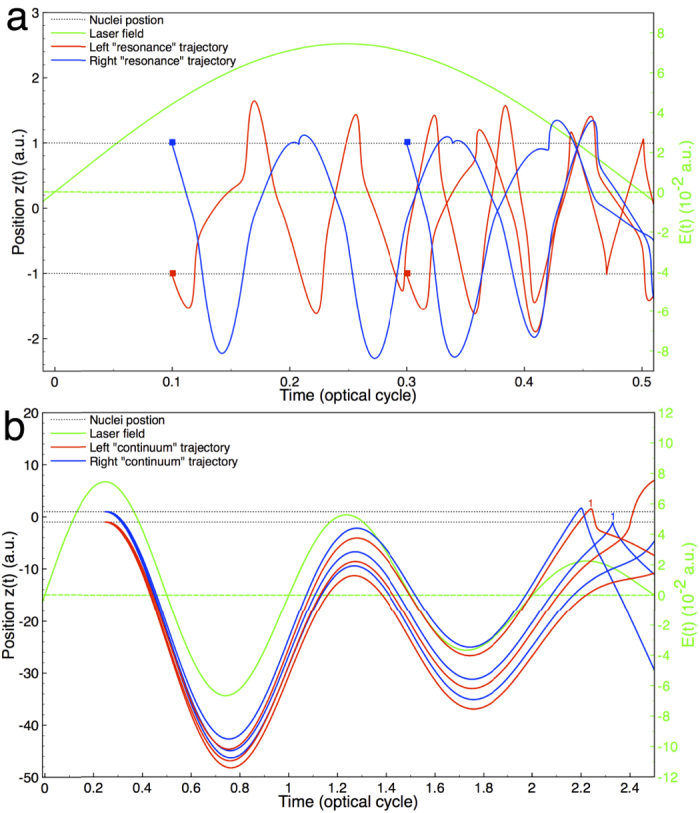
Semiclassical trajectories: Position and time, and scheme of resonance and continuum electron dynamics. (**a**) Several trajectories in below-threshold HHG (resonant). The red and blue squares are the corresponding time of release for the electron from either the right (blue) or left (red) hydrogen core. (**b**) Several trajectories in above-threshold HHG (continuum). Here the initial condition is that the electrons with an initial velocity *v*_0_ are released ‘opposite’ to the electronic-field force **F**_*z*_ from the left and right hydrogen cores (*z* = ±1 a.u.). The green solid line indicates the corresponding laser field, and the laser parameters used are the same as those in Fig. 1.

To explain the detailed electronic dynamic behaviors in the below-, near-, and above-threshold harmonic generation, the positions (*z*) of the electrons as a function of time are shown in Figs 3c and 4a,b, respectively. Figure 3c shows the position of the electrons as a function of the time in the below-, near- and above-threshold regions; the corresponding initial condition is marked by the arrows in Fig. 3b. Initially the electrons with a small velocity are released along (or against) the laser field direction at either hydrogen cores (*z* = ±1 a.u.); here the minimum initial velocity is *v*_0_ = 0 and the maximum is the height of the barrier at the time of release, and typically the initial velocities used are |*v*_0_| ≤ 1.0 (in a.u.). Figure 3c presents several short trajectories (first return), long trajectories (first return), and multi-rescattering trajectories (second through third return) in below-, near- and above-threshold HHG along with the corresponding laser field.

For the long trajectories, the electron first tunnels through the lower part of the barrier potential (leaving the core near maximum of the laser field), accelerates and returns to the left (*z* = −1 a.u.) or right (*z* = 1 a.u.) sided hydrogen core. This is a typical tunneling process. Nevertheless, when the electron once again returns to the left or right sided hydrogen core, it now faces the combined molecule-field potential wall (the higher part of the barrier potential, HBP on the other side) and tunneling is unlikely. Thus the electron first moves towards the HBP and subsequently absorbs several photons to a higher energy state and quickly returns to the ground state, unless the return energy is greater than zero (marked by 1; black text). Such a mechanism is similar to the multiphoton process. The long-trajectory electrons have multiscattering behaviors, and the first return 1 has a travel time around 0.8–1.0 optical cycle. However, the multirescattering process marked by 2–5 have a longer travel time (~1.2 optical cycles). Indeed, to end the first return marked by 1 (red text; *E* = 0 and *v*_*x,y,z*_ = 0), the electrons still move along the electric field, which allows them to quickly return to the core at near the peak intensity in around ~0.25 optical cycles (marked by 2). To end the first return marked by 1 (black text; *E* > 0, |*v*_*z*_| > 1.2, and |*v*_*x*_| ≈ |*v*_*y*_| = 0.07 a.u.), the electrons have a large velocity moving along the electric field, which allows them to have a longer travel time, which they return to the core in around ~0.6 optical cycles (marked by 3). The short trajectory (first return) has a different mechanism, where the electron leaves the core near the zero laser fields and always faces the HBP, where tunneling ionization is impossible. As a result, the electron leaves the left sided hydrogen core (*z* = −1 a.u.) and collides with the right sided hydrogen core (*z* = 1 a.u.) within 0.15 optical cycles due to the molecular potential force exerted from the right hydrogen core. It is then pulled back to the core it was released from (left sided hydrogen core (*z* = −1 a.u.)) quickly by the molecular potential. This behavior only involves the multiphoton process. The short-trajectories electrons 1’ are allowed to quickly revisit the core with a short travel time (around 0.3 optical cycles), which nearly coincides with the local peak of the laser field (see the green solid line). The short trajectory dynamics of 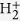 molecules involves mainly multiphoton process, which is different from that of the atomic cases, where the tunneling mechanism dominates^23^,^24^,^31^. Note we find that only the long trajectory electrons are associated with the multi-rescatterings observed in below-, near-, and above-threshold regions, and there corresponding travel time is larger than one optical cycle. To confirm that the multi-rescattering strongly contributes to the generation of the below- and near-threshold harmonics, as predicted by the semiclassical approach, we calculate the probability of the electrons with the corresponding return time t and return energy E as shown in Supplementary Fig. 1 by using an extended semiclassical method^33^ (Supplementary Method).

Furthermore, Fig. 4a shows several trajectories which are released from the left (*z* = −1 a.u.; red solid line) or right (*z* = 1 a.u.; blue solid line) sided hydrogen core and return almost immediately and/or oscillates between the left or right sided hydrogen core. This trajectory behavior correspond to the “resonant” or bounded (un-ionized) trajectories^24^,^34^. These “resonant” trajectories contribute to the near-resonance harmonic, namely the 7th harmonic, which coincides with the 1*σ*_*g*_–1*σ*_*u*_ multiphoton resonance-transition of 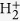. Lastly, Fig. 4b shows several trajectories which are released from the left or right sided hydrogen core and have a long travel time (>1.95 optical cycles) before returning to either the left or right hydrogen core. Also, we can say this trajectory behavior corresponds to the “continuum” trajectories. These “continuum” trajectories contribute only to the above-threshold harmonics and they can be attributed to the tunneling process. In Fig. 4b, several of the “continuum” trajectories first returns are rescattered off the opposite hydrogen core which they were released from, hence, the right and left trajectories 1. At 2.25 optical cycles the trajectory 1 rescatters off the right sided hydrogen core (*z* = 1 a.u.), and it was released some time before from the left sided hydrogen core (*z* = −1 a.u.). Also, at 2.32 optical cycles, the trajectory 1 rescatters off the left sided hydrogen core (*z* = −1 a.u.), and it was released some time before from the right sided hydrogen core (*z* = 1 a.u.).

### Dynamical phase of below-, near-, and above-threshold HHG

In this section we analyze and explore the dynamical phase of below-, near-, and above-threshold harmonic emission process. According to the time profile for each harmonic^35^, the time at the peak intensities suggest the emission times of such harmonic during the interaction of the 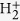 molecule with the applied laser field. In addition, the corresponding dynamical phases imply the underlying physical process that gives rise to such harmonic. The time profile 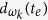 for a specific harmonic *ω*_*k*_ can be obtained by the reconstruction of the SST representation. The dynamical phase *ϕ*_*k*_(*t*_*e*_) is therein extracted from 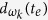 by 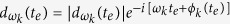.

**Figure 5:**
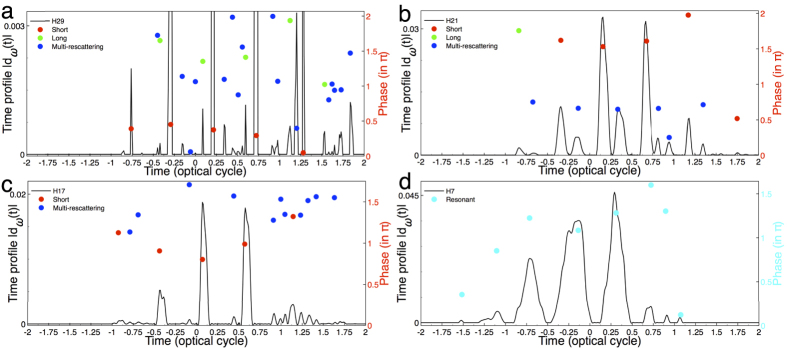
SST time profiles and dynamical phase of the harmonic spectra of 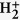. (**a**) The time profiles of the 29th harmonic (H29, above threshold). The red, green, and blue dots are the corresponding dynamical phases for the peak intensity of the short, long, and multi-rescattering trajectories calculated by the SST, respectively. (**b**) Same as a, for the time profiles of the 21st harmonic (H21, near threshold). (**c**) Same as a, for the time profiles of the 17th harmonic (H17, below threshold, nonresonant). (**d**) Same as a, for the time profiles of the 7th harmonic (H7, below-threshold resonant). The light blue dots are the corresponding dynamical phases of the resonant trajectories. The laser parameters used are the same as those in Fig. 1.

We first consider the time profile and dynamical phases of the 29th harmonic (H29) in Fig. 5a as an example of the harmonics in the above-threshold region, where the tunneling mechanism is dominant. As shown in the figure, there is a major peak followed by several small peaks within each half optical cycle of the time profile. By marking the dynamical phases of these peaks and comparing the corresponding emission times with those of the semiclassical trajectories in Fig. 3a,b, we find that the major peak corresponds to the short trajectories, while the minor ones correspond to the long trajectories and multi-rescattering trajectories. The time profile presents a complex pattern within each half optical cycle, suggesting that in addition to the short and long trajectories, the contribution of multi-rescattering trajectories become significant. In addition, the dynamical phase of the long (green dots) and multi-rescattering (blue dots) trajectories are sensitive to the laser profile (since the travel time for these trajectories are long), whereas those for the short trajectories (red dots) are not (shorter travel time). Similar behavior is observed in our previous atomic studies of the H-atom^36^, Na-atom^37^, and Cs-atom^24^.

Figure 5b shows the time profile of the 21st harmonic (H21), a representative case in the near-threshold regime. The time profile of the 21st harmonic (H21) presents a more structured pattern as to the above-threshold 29th harmonic (H29) in Fig. 5a. In Fig. 5b within each half optical cycle the dominant peak is also recognized as the short trajectory (green dots), despite its large phase (located between 1.5–2*π*). The multi-rescattering trajectories (blue dots) contribute to the generation of the harmonics in this region and is more dominate (greater intensity of 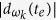) for the near-threshold 21st harmonic (H21) than any other harmonic orders in Fig. 5a,c. Also, the near-threshold 21st harmonic (H21) multi-rescattering trajectories is composed of mainly a single phase, indicating that the dynamical phases are locked. Similar behavior is also observed in the Cs-atom^24^ case recently studied. We observe that the envelope of the time profile resembles that of the laser profile. We find the multiple returns (multi-rescattering) have strong contributions to near-threshold harmonic generation (this can be seen in the SST representation Fig. 1 and in the semiclassical analysis Fig. 3a,b), and the multi-rescattering process depends on the intensity of the laser field as observed in the previous studies of atomic systems^23^,^24^,^31^. When the laser intensity is increased, the multi-rescattering behavior of the electrons becomes more pronounced. Similar to the results in H29, the dynamical phases for the short trajectories (green dots) are not sensitive to the laser profile. The dynamical phases for the near-threshold H21 multi-rescattering (blue dots) trajectories do differ from the above-threshold H29 multi-rescattering (blue dots), in which the H21 multi-rescattering trajectories are not sensitive to the laser profile and are almost constant during the emission process. The phase invariance leads to a zero chirp, thereby implying the multiphoton process is dominant, as explained in Fig. 3c.

Figure 5c shows the time profile of the below-threshold 17th harmonic (H17). Within each half optical cycle, the dominant peak is identified as the short trajectory (red dots) and the multi-rescattering (blue dots) trajectories still contribute to the generation of the harmonics in this below-threshold region. Here, we can say both the dynamical phases for the short (green dots) and multi-rescattering (blue dots) trajectories are relatively sensitive to the laser profile. The time profile for the 7th resonant harmonic (H7) is shown in Fig. 5d. We see that the envelope of the time profile resembles that of the laser profile. In the multiphoton regime, the probability of absorbing *N* photons is roughly proportional to *I*^*N*^ (*I* is the laser intensity)^28^,^38^. The major peaks are indicated as the resonant trajectories and their dynamical phases are denoted by light blue dots. Here, we see the dynamical phase for the multiphoton resonant harmonic is sensitive to the laser profile. This sensitivity is due to the complex oscillitory behavior of the electron between the left and right hydrogen cores which we observed for the “resonant” trajectories in Fig. 4a.

## Discussion

We have presented an 3D *ab initio* study of on the below-, near- and above-threshold harmonic generation of 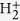 molecules in an intense NIR laser field by accurately solving the two-centered TDSE by means of the TDGPS method^25^. We have performed the quantum trajectories analysis of the below-, near- and above-threshold harmonics by using the SST time-frequency profiles and identified the trajectories with the assistance of an extended semiclassical simulation. Our 3D extended semiclassical results allow us to study electron trajectories as a function of energy, time, and position to gather dynamics for each of the individual hydrogen cores of the 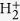 molecule. We find that multiphoton-dominated short trajectories, long trajectories, multi-rescattering trajectories and resonant trajectories in the below- and near-threshold HHG involve only the electron scattered off the combined molecule-field potential wall followed by the absorption of photons. In particular, we find that only the multi-rescattering trajectories for the near-threshold H21 harmonic possess the single phase-component, which implies that the dynamical phases in near-threshold multi-rescattering harmonic generation are locked. Furthermore, we find that the below-, near- and above-threshold harmonic generation represents a complex physical processes where all kinds of trajectories contribute to HHG. However, the effects of multi-rescattering trajectories gradually disappear in the lower-order harmonic generation. Our study enables us to obtain a deeper understanding of the novel mechanisms of below-, near-, and above-threshold harmonic generation for diatomic molecules for the first time.

## Methods

### Time-dependent Schrödinger equation

The 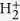 molecules HHG power spectra can be investigated accurately and efficiently by solving the 3D TDSE in space and time by means of the time-dependent generalized pseudospectral method (TDGPS)^25^ in prolate spheroidal coordinates^38^,^39^,^40^. In addition, we present the high-precision bound state energies of 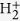 by means of the GPS method in prolate spheroidal coordinates (Supplementary Table 1). Once the time-dependent wave function is available, we can calculate the expectation value of the induced dipole moment in acceleration form, and the HHG power spectra can be obtained by the Fourier transform of the time-dependent dipole acceleration.

### SST time-frequency analysis

In this work, we perform the time-frequency analysis of the harmonic spectra of 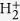 by means of the SST method. The SST is proposed to address the intrinsic blurring in the linear type time-frequency methods, such as the Gabor transform and Wavelet transform, and the accuracy of the SST technique are well supported by mathematical analysis.

### Semiclassical method

To understand the quantum-trajectory characteristics, we have extended the semiclassical three-step model by including the accurate electronic structure information in the selective analysis of the quantum trajectories in the below-, near-, and above-threshold generation of 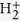 diatomic molecule. The initial condition is that the electrons are released at either the left or right hydrogen core with the initial velocity along or opposite the polarization direction of the laser field. In our calculation, the semiclassical results are obtained by solving the Newton’s equation including the molecular potential, which is given by




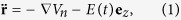




where *E(t*) is the electric field strength of the laser field and *V*_*n*_ is the Coulomb interaction with the nuclei (the charge of each center *Z* is unity) defined by:




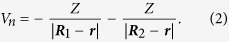




## Additional Information

**How to cite this article**: Heslar, J. and Chu, S.-I. Unravelling the dynamical origin of below- and near-threshold harmonic generation of 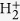 in an intense NIR laser field. *Sci. Rep.* **6**, 37774; doi: 10.1038/srep37774 (2016).

**Publisher’s note:** Springer Nature remains neutral with regard to jurisdictional claims in published maps and institutional affiliations.

## Supplementary Material

Supplementary Information
