# Supplementary material for: Unravelling the dynamical origin of below- and near-threshold harmonic generation of H2+ in an intense NIR laser field
Source: Sci Rep. 2016 Nov 24;6:37774. doi: 10.1038/srep37774 (PMC5121898; doi:10.1038/srep37774)
Supplement: Supplementary Information [file srep37774-s1.pdf]

Unravelling the dynamical origin of below- and near-threshold harmonic generation of H_2^+ in an intense NIR laser field

John Heslar[1] and Shih-I Chu[1,2,*]

[1]*Center for Quantum Science and Engineering, and Center for Advanced Study in Theoretical Sciences, Department of Physics, National Taiwan University, Taipei 10617, Taiwan*

[2]*Department of Chemistry, University of Kansas, Lawrence, Kansas 66045, USA*

* Correspondence and requests for materials should be addressed to S. I. C. (email: sichu@ku.edu)

SUPPLEMENTARY FIGURES

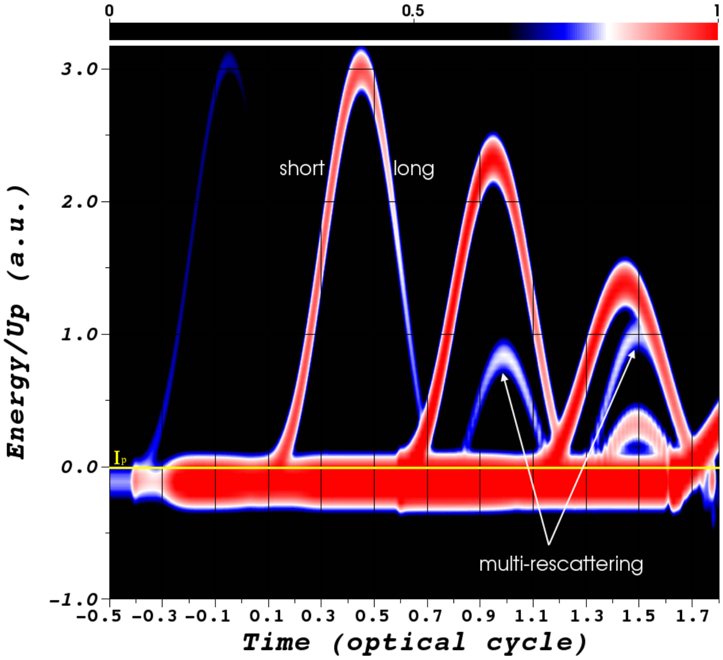

FIG. 1. Supplementary Figure 1: Semiclassical returning energy map. Note that the probability of the electrons with the corresponding return time and return energy is calculated by using an extended semiclassical method introduced in Ref. [1]. The yellow solid line indicates the ionization potential I_p.

SUPPLEMENTARY TABLES

TABLE I. High-precision bound state energies of H_2^+ at internuclear separation 2 a.u.

State	Energy (a.u.)
$1\sigma_g$	-1.1026342144949
$1\sigma_u$	-0.6675343922023
$1\pi_u$	-0.4287718198958
$2\sigma_g$	-0.3608648753395
$2\sigma_u$	-0.2554131650864
$3\sigma_g$	-0.2357776288255
$1\pi_g$	-0.2266996266436

SUPPLEMENTARY METHODS

Ab initio Simulation of the High-order Harmonic Generation Spectra of the H_2^+ Diatomic Molecule

Detailed numerical procedures can be found in Refs. [2–7]. The time-dependent electron wave function $\psi(\boldsymbol{r},t)$ of H_2^+ at a fixed internuclear distance satisfies the TDSE (atomic units $\hbar = m = e = 1$ are used unless stated otherwise):

$$i\frac{\partial}{\partial t}\psi(\boldsymbol{r},t) = \left[H_0(\boldsymbol{r}) + V_{\mathrm{ext}}(\boldsymbol{r},t)\right]\psi(\boldsymbol{r},t). \tag{1}$$

Here $H_0(\boldsymbol{r})$ is the unperturbed electronic Hamiltonian:

$$H_0(\boldsymbol{r}) = -\frac{1}{2}\nabla^2 + V_{\mathrm{n}}(\xi,\eta). \tag{2}$$

$V_{\mathrm{n}}(\xi,\eta)$ being the Coulomb interaction with the nuclei (the charge of each center is unity),

$$V_{\mathrm{n}}(\xi,\eta) = -\frac{2\xi}{a(\xi^2-\eta^2)}. \tag{3}$$

Here a is a half internuclear separation ($a = R/2$); the nuclei are located at the points $-a$ and a on the z axis. The equilibrium internuclear separation for the H_2^+ diatomic molecule is $R = 2$ a.u.

The H_2^+ molecules high-order harmonic generation (HHG) power spectra can be investigated accurately and efficiently by solving the 3D TDSE in space and time by means of the time-dependent generalized pseudospectral method (TDGPS) [8] in prolate spheroidal coordinates [3, 4, 9]. Once the time-dependent wave function $\psi(\xi,\eta,t)$ is available, we can calculate the expectation value of the induced dipole moment in acceleration form :

$$d_A(t) = <\psi(\xi,\eta,t)|\nabla V_{\mathrm{n}}(\xi,\eta)|\psi(\xi,\eta,t)> - E(t). \tag{4}$$

The HHG power spectra $S(\omega)$ (spectral density of the radiation energy) in the acceleration form can be obtained by the Fourier transformation of time-dependent dipole moment in acceleration form $d_A(t)$,

$$S(\omega) = \frac{2}{3\pi c^3}\left|\int_{-\infty}^{\infty} d_A(t)\exp(i\omega t)dt\right|^2. \tag{5}$$

Synchrosqueezing Transform

We perform the time-frequency analysis on the induced dipole moment in acceleration form $d_A(t')$ of the H_2^+ diatomic molecule interacting with the applied laser field by means of the synchrosqueezing transform (SST) [10, 11]. The SST is described as:

$$S(t,\xi) = \int \frac{1}{\sqrt{\omega}}V(t,\omega)\frac{1}{\alpha\sqrt{\pi}}\exp\left(-\left[\frac{\xi-\Omega_f(t,\omega)}{\alpha}\right]^2\right)d\omega, \tag{6}$$

where $V(t,\omega)$ is the Morlet wavelet transform, $\Omega_f(t,\omega)$ is the reallocation rule function, and α is a smoothing parameter. In this study, $\alpha = 2.6$. The Morlet wavelet transform is given as:

$$V(t,\omega) = \int d_A(t')\sqrt{\omega}W(\omega(t'-t))dt', \tag{7}$$

where

$$W(x) = \frac{1}{\sqrt{\tau}}\exp(ix)\exp\left(-\frac{x^2}{2\tau^2}\right) \tag{8}$$

is the mother wavelet. The reallocation rule function is defined as:

$$\Omega_f(t,\omega) = \begin{cases} \dfrac{-i\partial_t V(t,\omega)}{V(t,\omega)} & \text{for } V(t,\omega) \neq 0 \\ \infty & \text{for } V(t,\omega) = 0 \end{cases}, \tag{9}$$

where ∂_t denotes the partial derivative in the temporal axis.

The time profile $d_{\omega_k}(t_e)$ for some harmonic ω_k from the SST analysis can be obtained from the reconstruction function:

$$d_{\omega_k}(t_e) = \Re\left\{\frac{1}{R_W}\int_{\xi_1}^{\xi_2}\frac{S(t,\xi)}{\sqrt{\omega}}d\omega\right\}, \tag{10}$$

where (ξ_1, ξ_2) is the neighborhood of such harmonic, $R_W = \int \frac{\widehat{W}(\eta)}{\eta}d\eta$ and $\widehat{W}(\eta)$ is the Fourier transform of $W(x)$, and $\Re$ denotes the real part.

Extended Semiclassical Method

In Supplementary Figure 1 we calculate the probability of the electrons with the corresponding return time t and return energy E, by using an extended semiclassical method [1], which can be obtained from the following expression:

$$\begin{aligned}\frac{dP(E,t)}{dEdt} = &\int dt'd\mathbf{v}W(|E(t')|)P(\mathbf{v})C_t(t',\mathbf{r}_0,\mathbf{v},E_r,t_r)\\ &\times \delta(E-E_r)\delta(t-t_r),\end{aligned} \tag{11}$$

where $W(|E(t')|)$ is the instantaneous tunneling ionization rate, E_r and t_r are the returning time and returning energy (kinetic energy + potential energy) for given trajectories, and $P(\mathbf{v})$ is the Gaussian initial velocity distributions. Each trajectory is monitored for all the approaches to either of the hydrogen cores ($z = \pm 1$ a.u.) for the H_2^+ molecule. If an electron trajectory is such that it can return to either of the hydrogen cores at time t_r with a returning energy E_r, the factor $C_t(t',\mathbf{r}_0,\mathbf{v},E_r,t_r)$ is set to 1. Otherwise, $C_t(t',\mathbf{r}_0,\mathbf{v},E_r,t_r) = 0$. In Supplementary Figure 1, it is clearly seen that several multirescattering trajectories are superposed after the peak of laser field.

---

[1] Carrera, J. J., Tong, X. M. & Chu, S. I. Creation and control of a single coherent attosecond xuv pulse by few-cycle intense laser pulses. *Phys. Rev. A* **74**, 023404 (2006).
[2] Telnov, D. A. & Chu, S. I. Time-dependent generalized pseudospectral method for accurate treatment of multiphoton processes of diatomic molecules in intense laser fields. *Comput. Phys. Comm.* **182**, 18 (2011).
[3] Heslar, J., Telnov, D. A. & Chu, S. I. Enhancement of vuv and euv generation by field-controlled resonance structures of diatomic molecules. *Phys. Rev. A* **93**, 063401 (2016).
[4] Telnov, D. A., Heslar, J. & Chu, S. I. Effect of nuclear vibration on high-order harmonic generation of aligned h_2^+ molecules. *Phys. Rev. A* **90**, 063412 (2014).
[5] Avanaki, K. N., Telnov, D. A. & Chu, S. I. Above- and below-threshold high-order-harmonic generation of h_2^+ in intense elliptically polarized laser fields. *Phys. Rev. A* **90**, 033425 (2014).
[6] Avanaki, K. N., Telnov, D. A., Jooya, H. Z. & Chu, S. I. Generation of below-threshold even harmonics by a stretched h_2^+ molecular ion in intense linearly and circularly polarized laser fields. *Phys. Rev. A* **92**, 063811 (2015).
[7] Avanaki, K. N., Telnov, D. A. & Chu, S. I. Exploration of the origin of anomalous dependence for near-threshold harmonics in h_2^+ on the ellipticity of driving laser fields. *J. Phys. B* **49**, 114002 (2016).
[8] Tong, X. M. & Chu, S. I. *Chem. Phys.* **217**, 119 (1997).
[9] Chu, X. & Chu, S. I. Complex-scaling generalized pseudospectral method for quasienergy resonance states in two-center systems: Application to the floquet study of charge resonance enhanced multiphoton ionization of molecular ions in intense low-frequency laser fields. *Phys. Rev. A* **63**, 013414 (2001).
[10] Chen, Y. C., Cheng, M. Y. & Wu, H. T. Non-parametric and adaptive modelling of dynamic periodicity and trend with heteroscedastic and dependent errors. *J. R. Stat. Soc. Ser. B Stat. Methodol.* **76**, 651 (2014).
[11] Sheu, Y. L., Hsu, L. Y., Wu, H. T., Li, P. C. & Chu, S. I. A new time-frequency method to reveal quantum dynamics of hydrogen in intense laser pulses: synchrosqueezing transform. *AIP Adv.* **4**, 117138 (2014).
